# Adherence to Indonesia's Dietary Guidelines Among Lactating Women: Insights for Policy and Practice

**DOI:** 10.1111/mcn.70075

**Published:** 2025-07-29

**Authors:** Sofa Rahmannia, Kevin Murray, Gina Arena, Aly Diana, Rosalind Gibson, Siobhan Hickling

**Affiliations:** ^1^ School of Population and Global Health The University of Western Australia Crawley Western Australia Australia; ^2^ Faculty of Medicine Universitas Pasundan Bandung West Java Indonesia; ^3^ Medical School The University of Western Australia Nedlands Western Australia Australia; ^4^ The Kids Research Institute Nedlands Western Australia Australia; ^5^ Nutrition Working Group, Faculty of Medicine Universitas Padjadjaran Bandung West Java Indonesia; ^6^ Department of Public Health, Faculty of Medicine Universitas Padjadjaran Bandung West Java Indonesia; ^7^ Department of Human Nutrition University of Otago Dunedin New Zealand

**Keywords:** dietary guidelines, growth, guideline adherence, human milk, Indonesia, lactation, micronutrients, nutritional status

## Abstract

This study investigated adherence to Indonesia's Dietary Guidelines (IDG) among lactating women, examining related factors and association with nutrient intake adequacy, maternal and infant biomarkers, body mass index, and growth. Participants were lactating women (*n* = 220) from urban and rural West Java, Indonesia. Dietary intake (via 3‐day weighed food records), anthropometry and blood samples were assessed. Adherence was evaluated using a scoring system tailored for IDG and adapted from the Healthy Eating Index to assess intake of food groups, sugar, salt, fat, water, coffee, and breakfast habits. Starchy staples intake exceeded recommendations by nearly double (median 7.1 vs. recommended 3–4 servings/day), while vegetable (0.5 servings/day), fruit (1.0), and water (1300 mL/day) intake fell notably short. Protein‐rich food intake (3.5 servings/day) was closer to target. Only 1% of participants met three out of four food group targets. Adherence to the meal‐based *MyPlate* framework showed similar imbalances, with 68% of the plate occupied by starchy staples versus the recommended 33%. Sociodemographic factors, including education, wealth, and family size, were associated with adherence to IDG components. For instance, women in the highest wealth quintile had higher adherence scores for starchy staple moderation (mean 4.3) than those in the lowest (mean 2.9). Adherence to IDG components correlated positively with nutrient intake adequacy (e.g. protein‐rich food and overall adequacy: *r* = 0.19, 95% CI: 0.06–0.32) but not consistently with maternal or infant biomarkers. These findings highlight the need to refine dietary guidelines with clearer portion guidance and consideration of factors beyond intake adequacy during lactation.

## Summary

- Consumption of starchy staples, particularly white rice, often exceeds recommendations, highlighting the need for clearer portion guidance.
- Sociodemographic factors, including education, economic status, and family size are associated with adherence to staple recommendations and water intake.
- Fruit and vegetable adherence remains low across sociodemographic groups.
- Protein‐rich food adherence is high across sociodemographic groups and associated with nutrient adequacy but not biomarkers, warranting further research on differences between animal‐ and plant‐based proteins.
- Given low median intake observed and no association with breastmilk volume, the 3000 mL/day water intake guideline may merit further evaluation in lactating populations.

## Graphical abstract

Most lactating women in Indonesia consume excessive starchy staples and insufficient vegetables, fruits, and water, while protein‐rich food intake is closer to recommendations. Overall dietary adherence improves nutrient adequacy but does not consistently translate into better nutritional biomarkers or growth outcomes, highlighting the need to refine lactation‐specific dietary recommendations.

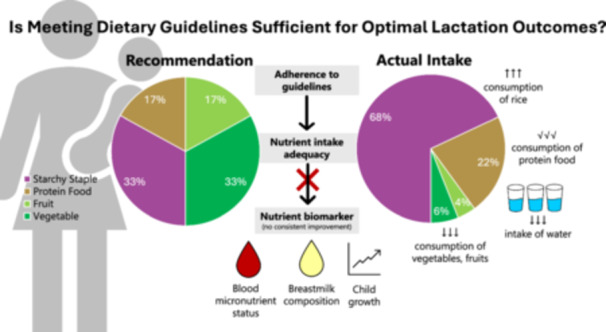

## 1 Introduction

Optimal nutrition during lactation is essential for maternal and infant health, supporting milk production, maternal recovery, and overall well‐being. Nutrient demands increase substantially during this period, particularly for key micronutrients such as iron, zinc, vitamin A, and vitamin B12, which are vital for immune function, cognitive development, and red blood cell production in both mothers and infants (Allen et al. 2018; Kodentsova et al. 2021). These four micronutrients are also among the most commonly deficient in low‐ and middle‐income countries (LMICs), including Indonesia (Daniels et al. 2019; Liu et al. 2022).

Despite the critical role of these micronutrients in maternal health and their risk of deficiency, dietary guidelines in many countries, including Indonesia's Dietary Guidelines (IDGs), are primarily designed for the general population and heavily oriented toward non‐communicable disease (NCD) prevention, such as obesity, hypertension, and cardiovascular disease, rather than maternal or lactation‐specific outcomes (Locke et al. 2018; Ministry of Health Republic Indonesia 2014; Williams et al. 2021). It remains unclear whether these general recommendations adequately address the unique nutritional demands of lactating women, particularly concerning micronutrient status, postpartum weight, and infant growth (Rahmannia et al. 2024). In Indonesia, diets are typically dominated by white rice, with relatively low and irregular consumption of animal‐source foods, fruits, and vegetables. Cultural norms, economic limitations, and meal structuring practices often prioritise staple energy sources over nutrient diversity (Colozza and Avendano 2019). Investigating the association between adherence to dietary recommendations and both nutrient adequacy and nutritional biomarkers can provide essential information for evaluating and refining Food‐Based Dietary Guidelines (FBDGs) to ensure they better support maternal and infant health (Food and Agriculture Organization of the United Nations FAO 2024).

Research on dietary adherence during lactation is also limited, especially in LMIC settings. Most studies have focused on high‐income countries or target other life stages, such as pregnancy or early childhood (Ferranti et al. 2019; Filatava et al. 2024). Little is known about adherence to the FBDGs during lactation and their impact on postpartum weight, maternal and infant nutrition, or growth in LMICs where dietary behaviours are frequently influenced by food access, cultural practices, and socioeconomic disparities (Rahmannia et al. 2024).

The IDGs recommend daily servings of starchy staples (3–4 servings/day), vegetables (3–4 servings/day), fruits (2–3 servings/day), and protein‐rich foods (2–4 servings/day) while limiting sugar, salt, and fat intake for the general population. They also promote regular hydration and healthy eating habits, such as having breakfast. The IDGs also advise increased variety and quantity for lactating women, although recommendations for portion sizes are not specified. To provide practical guidance on meal composition, the MyPlate (*Isi Piringku*) model, derived from the IDGs, offers a structured framework for balanced nutrient intake. It translates these recommendations into meal‐based visual guidance, suggesting one‐third of the plate for starchy staples, one‐third for vegetables, and one‐sixth each for protein‐rich foods and fruits (Ministry of Health Republic Indonesia 2014).

Given the unique nutrient demands during lactation and the absence of lactation‐specific dietary guidance, this study aims to (1) assess adherence to IDGs and MyPlate among Indonesian lactating women, (2) identify sociodemographic factors influencing adherence, (3) investigate the relationship between adherence scores and usual nutrient intake, assess whether higher adherence aligns with nutrient adequacy, and (4) explore associations between adherence to food group recommendations and nutritional status, including maternal BMI, maternal and infant blood micronutrient biomarkers (iron, zinc, vitamin A, and B12), breastmilk composition, and infant growth. By addressing these objectives, this study aims to determine whether existing guidelines are compatible with the nutritional needs of lactating women and provide evidence to inform the refinement of FBDGs for this population in Indonesia and similar LMICs contexts.

## 2 Methods

### 2.1 Study Design and Population

This study is part of the Indonesian breastfeeding cohort study, which tracked lactating women and their infants for the first 2 years of life (Gibson et al. 2020; Liu et al. 2019). Women were recruited between 2017 and 2019 using attendance records from routine postnatal visits to *posyandu* (community health posts), with the assistance of local community health cadres. Participants were recruited from both urban (Bandung, *n* = 113) and rural (Sumedang, *n* = 107) areas in West Java, Indonesia, by local community health cadres. The sample size for this study (*n* = 220) was derived from the parent cohort study, which aimed to distinguish exclusively breastfed (EBF) from partially breastfed (PBF) infants during the first 6 months of life. It was calculated that 100 mother–infant pairs per group would provide sufficient power, accounting for dropout, and is adequate to support the multivariable regression analyses used in this investigation. Inclusion criteria required women to be without chronic diseases or acute malnutrition, have given birth to a full‐term infant weighing at least 2500 g, and be breastfeeding.

### 2.2 Sociodemographic Characteristics

Sociodemographic data were collected through an interviewer‐administered questionnaire at 2 months postpartum (±1 week). A wealth index was established using principal component analysis based on household assets, following the Demographic and Health Surveys guidelines (Fry et al. 2014).

### 2.3 Maternal Dietary Assessment

Dietary intake was assessed over three nonconsecutive days for all participants. In rural areas, trained cadres conducted 12‐h in‐home food weighing due to limited literacy and capacity among participants to conduct self‐recording, while in urban areas, mothers were trained to weigh and record their own food and beverage intake using digital scales provided by the investigators. Data collection was staggered across the calendar year, with participants recruited throughout both dry and rainy seasons, including during the Ramadan fasting period. This distribution helped minimise systematic seasonal bias. Supplement use was recorded through the dietary records. Foods were categorised into starchy staples (e.g., rice, noodles), vegetables, fruits, and protein‐rich foods. Coffee intake was recorded from food records, while drinking water intake included plain water and beverages (tea, juice, milk) but excluded water from foods.

Nutrient intakes were estimated using the Indonesian Food Composition Table (FCT), incorporating mandatory wheat flour fortification with thiamine, riboflavin, iron, zinc, and folic acid. Salt intake was estimated from intrinsic sodium (FCT) and discretionary salt in cooking based on standard recipes. Sugar intake included natural sources (fruits, dairy), added sugars in processed foods, and additional sugar in homemade foods and beverages. Fats and oils were derived from intrinsic fat content and cooking oils, calculated using recipes.

### 2.4 Adherence to Meal‐Basis Recommendation: MyPlate (*Isi Piringku*)

To assess adherence to the MyPlate (*Isi Piringku*) guidelines, the total intake (grams per meal) of starchy staples, protein‐rich foods, vegetables, and fruits was calculated and expressed as proportions of the total food intake per meal. These proportions were then averaged across all meals for the 3 days for each participant. A scoring system was developed to assess adherence, comparing the observed intake proportions per meal to MyPlate recommendations, which allocate one‐third (33%) of the plate to starchy staples, one‐third (33%) to vegetables, one‐sixth (17%) each to protein‐rich foods and fruit.

### 2.5 Adherence to Indonesian Dietary Guidelines Score Determination and Calculation

We reviewed the IDG and specific recommendations for lactating women to determine the scoring system (Ministry of Health Republic Indonesia 2014). Adherence was assessed using a scoring system adapted from the Healthy Eating Index (HEI) 2020 (Krebs‐Smith et al. 2018; Shams‐White et al. 2023). The HEI scoring method was chosen because it generates continuous adherence values, allowing for a more detailed analysis of how adherence levels relate to nutrient adequacy and nutritional biomarkers. Ten dietary components were measured, aligned with IDG recommendations, and adjusted for lactating women. The IDG suggests 3–4 servings/day for starchy staples and vegetables, 2–3 servings/day for fruits, and 2–4 servings/day for protein‐rich foods that apply to all populations. Guidelines for salt (sodium) (< 2000 mg), sugar (< 0% energy), and fat (< 25% energy) follow global standards (World Health Organization 2012, 2015). During lactation, the IDG allows up to three cups of coffee daily and recommends a daily water intake of 3000 mL/day. A daily breakfast is generally recommended for all individuals (Ministry of Health Republic Indonesia 2014).

The scoring system, with a maximum of 100 points, assessed intake per 1000 kcal or as a percentage of energy intake. Each component carried equal weight (10 each), with scores proportionally assigned between minimum and maximum standards.

Components of the IDGs were divided into two groups for scoring: adequacy and moderation. Adequacy scoring was applied to the consumption of vegetables, fruits, protein‐rich foods, and water, with a minimum score assigned for no consumption and a maximum score when intake met the recommended level. For components with recommendation ranges, the highest value within the range was used to determine the maximum score to reflect the higher energy demands during lactation (Ministry of Health Republic Indonesia 2014).

Moderation scoring was used for starchy staples, sugar, salt, fats and oils, and coffee. Starchy staples were placed in this group because the IDG does not distinguish between whole and refined grains, and here, only 1% of all starchy staples consumed were whole grains. For moderation components, a minimum score was assigned if intake exceeded the recommendation. For starchy staples, intakes exceeding 5.7 servings per 1000 kcal were assigned a minimum score. This threshold was determined by adopting the HEI‐2020 refined grain scoring system. In the US dietary guidelines, the minimum score is assigned when intake exceeds 4.3 servings per 1000 kcal, corresponding to a daily maximum of three servings. To align this with the Indonesian recommendation of four servings per day, the threshold was proportionately adjusted to 5.7 servings per 1000 kcal. For fats and oils, salt and coffee, the minimum score was twice the recommended level, whereas, for sugar, it was four times the recommended level, an approach adapted from the HEI‐2020 scoring system (Krebs‐Smith et al. 2018; Shams‐White et al. 2023). The maximum score for moderation components was given when intakes were below the recommended cut‐off level (Ministry of Health Republic Indonesia 2014).

Daily breakfast was defined as a meal consumed between waking and 9:00 AM (Ministry of Health Republic Indonesia 2014). The maximum score for breakfast was achieved if participants had breakfast on all 3 days of a nonconsecutive 3‐day diet record.

### 2.6 Assessment of Nutrient Adequacy

The multiple source method (Harttig et al. 2011) was applied to estimate the usual intake of energy and nutrients for the study population. The probability of nutrient adequacy (PA) associated with the usual intake for each micronutrient and individual was then calculated using Estimated Average Requirements (EARs) from WHO/FAO, except for iron, calcium, and zinc. For iron and calcium, the EARs from IOM were applied (Institute of Medicine IOM 2001; Ross et al. 2011), assuming 10% bioavailability for iron reflecting mixed rice‐based diets of non‐menstruating lactating women (Institute of Medicine [IOM] 2001). For zinc, the International Zinc Nutrition Consultative Group (IZiNCG) EAR was used, assuming 34% bioavailability (Brown et al. 2004).

PA was assigned as 1 or 0 if the usual intake of the nutrient was ≥ or below the corresponding EAR. The Total PA Score, summing PAs across all nutrients, provided an overall measure of nutrient adequacy for each participant.

### 2.7 Analysis of Nutritional Biomarkers

At 5 months postpartum (±1 week), anthropometric measurements, blood, and breast milk samples were collected in the community health centre. Blood was drawn by trained phlebotomists, separated, and serum stored at −20°C until analysis (Centre de toxicologie du Québec [CTQ] 2021). Serum biomarkers, including ferritin, retinol‐binding protein (RBP), C‐reactive protein (CRP), and α−1‐acid glycoprotein (AGP), were measured via sandwich enzyme‐linked immunosorbent assay (Erhardt et al. 2004). Serum vitamin B12 was assessed via electrochemiluminescence immunoassay (Roche Diagnostics, GmbH), and serum zinc via inductively coupled plasma mass spectrometry (Agilent 7500 ICP‐MS) at the Centre for Trace Element Analysis, University of Otago, New Zealand. Ferritin, RBP, and zinc were adjusted for inflammation using the BRINDA regression approach (Suchdev et al. 2016), and serum zinc was further adjusted for time of day and the time since the last meal before collection (Arsenault et al. 2011).

Breastmilk intakes at 5 months were measured via the deuterium oxide dose‐to‐mother technique (Gibson et al. 2020). Breastmilk from one full breast was collected in the morning using a breast pump under strict contamination precautions, aliquoted into acid‐washed containers, and stored at −80°C. Breastmilk iron and zinc were analysed via ICP‐MS at the University of Otago, while vitamin B12 was assessed via chemiluminescent immunoassay at the USDA WHNRC, USA. Retinol and provitamin A carotenoids were measured via high‐performance liquid chromatography at WHNRC, USA. Detailed collection and micronutrient analysis procedures are described elsewhere (Daniels et al. 2019).

### 2.8 Assessment for Covariates: Infant Intakes and Anthropometric Status

Infant dietary intake was recorded alongside maternal diet. For EBF infants, nutrient intake was estimated based on breastmilk volume measured using the deuterium oxide dose‐to‐mother technique and its analysed nutrient composition. For PBF infants, total energy and micronutrient intake were calculated by combining nutrient intake from complementary foods, assessed via 3‐day weighed food records, with the estimated intake from breastmilk.

Infant weight and length were measured by trained research assistants using calibrated digital weighing scales (precision ± 10 g) and infant length boards (precision ± 1 mm) following standardised anthropometric procedures (de Onis et al. 2004). *Z*‐scores for weight‐for‐age (WAZ), length‐for‐age (LAZ), and weight‐for‐length (WLZ) were calculated using the WHO Child Growth Standards (WHO Multicentre Growth Reference Study Group 2006). Measurements were taken during the same visit as biological sample collection (at 5 months postpartum).

### 2.9 Statistical Analyses

Data were analysed using Stata 14.2. Adherence scores were reported as means with standard deviations, while the proportions of participants meeting recommendations were presented as percentages. Associations between adherence scores and sociodemographic characteristics were examined using multiple linear regression. Trends across ordinal variables were assessed using linear regression, treating categories as continuous variables.

Pearson correlation coefficients were calculated to assess associations between adherence scores for each dietary component and nutrient PA scores. Multiple linear regression models were used to evaluate associations between maternal adherence to dietary guidelines and various nutritional outcomes. These outcomes included maternal BMI, serum biomarkers (iron, zinc, RBP, vitamin B12), breastmilk composition (iron, zinc, retinol, vitamin B12), infant anthropometry (WAZ, HAZ, WHZ), and infant serum biomarkers (iron, zinc, vitamin A, and B12). Beta coefficients (*β*) with 95% confidence intervals (CIs) were reported to indicate the strength and direction of associations. A positive *β* value represents an increase in the outcome variable with higher adherence, while a negative *β* value represents a decrease.

For maternal outcomes, adjustments were made for maternal age, total energy intake, and nutrient‐specific intakes. Breastmilk composition models were further adjusted for breastmilk volume and exclusive breastfeeding status.

Infant growth models accounted for exclusive breastfeeding status, birth size (length and weight), inflammation markers (CRP), and energy intake. Infant biomarker models were adjusted for infant nutrient intake, maternal energy intake, and nutrient‐specific intakes.

Maternal and infant ferritin and RBP biomarker values were pre‐adjusted for inflammation (CRP, AGP), while maternal serum zinc values were adjusted for collection time. Breastmilk iron, zinc, and vitamin A were pre‐adjusted for milk volume and breastfeeding status.

All assumptions for multiple linear regression, including normality, multicollinearity, and homoscedasticity, were assessed, and no concerning violations were observed. A heatmap visualised correlations between adherence scores and nutritional biomarkers. Positive correlations were shown in blue, and negative in red. Multivariable regression included all adherence scores as predictors, with effect sizes unstandardised since adherence scores were on the same scale.

### 2.10 Ethics statement

This study was approved by the Human Research Ethics Committee (HREC) at the University of Western Australia (Approval Number: 2022/ET000721) and the Human Research Ethics Committee, Faculty of Medicine, Universitas Padjadjaran, Bandung, Indonesia (05/UN6.C1.3.2/KEPK/PN/2017). All participants provided written informed consent before their involvement in the study. The research adhered to the ethical principles outlined in the Declaration of Helsinki and followed the guidelines for the ethical treatment of human subjects.

## 3 Results

### 3.1 Adherence to Dietary Guidelines

**Figure 1:**
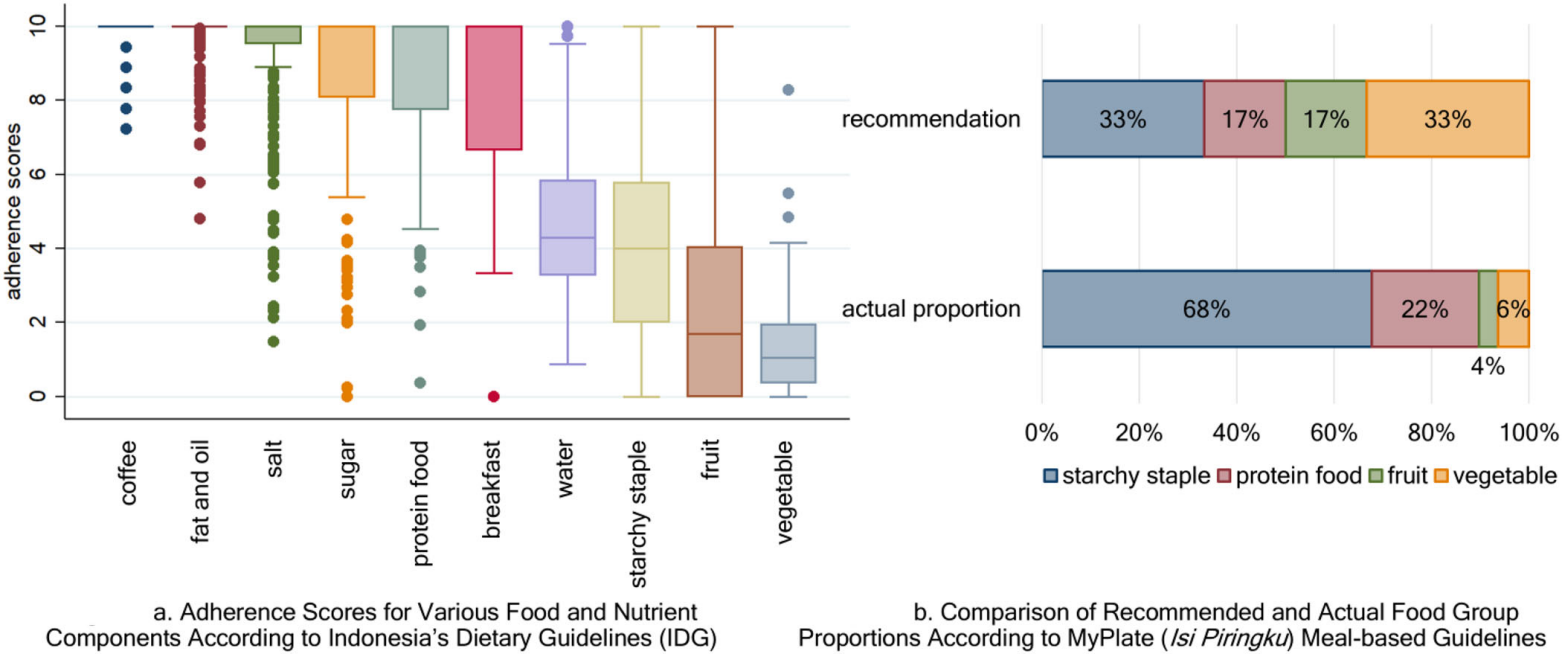
Adherence to dietary guidelines and MyPlate (*Isi Piringku*) proportions. (a) Adherence scores to the Indonesian Dietary Guidelines (IDG) for various food groups. Box plots indicate median adherence and variability across participants. (b) Comparison between recommended and actual proportions (%) of food groups based on MyPlate (*Isi Piringku*).

Table 1 presents the scoring system, actual intake, adherence scores, and the proportion of participants meeting or exceeding recommendations. Figure 1a shows adherence score distribution. On average, adherence to IDG was 68/100, with notable variations. Low adherence was observed for vegetables, fruits, starchy staples, and water, while protein‐rich foods, regular breakfast, and limits on coffee, sugar, salt, fats, and oils showed moderate to high adherence. No participants fully met all four primary food group recommendations (starchy staples, vegetables, fruits, and protein‐rich foods), while only 1% met three, 5% met two, and fewer than 50% met at least one (data not shown).

**Table 1:** Dietary recommendations, adherence scoring system, and intake distribution compared to Indonesia's Dietary Guidelines (IDG) among lactating women.

Component	Unit	Recommendation	Scoring system		Intake	Score	Under	Meeting	Over
		(2000 kcal)	Min	Max	median (Q1, Q3)	mean (SD)	*n* (%)	*n* (%)	n (%)
Vegetables^a^	serving	3–4	0	≥ 2/1000 kcal	0.5 (0.3, 0.9)	1.8 (1.5)	220 (100)	1 (0)	0 (0)
Fruits^a^	serving	2–3	0	≥ 1.5/1000 kcal	1 (0, 1.8)	4 (3.6)	175 (79)	31 (14)	15 (7)
Protein‐rich foods^a^	serving	2–4	0	≥ 2/1000 kcal	3.5 (2.6, 5)	8.9 (1.8)	20 (9)	105 (48)	96 (43)
Starchy staples^b^	serving	3–4	≥ 5.7/1000 kcal	≤ 2/1000 kcal	7.1 (5.7, 9)	3.9 (2.5)	2 (1)	14 (6)	205 (93)
Sugar^b^	%energy	≤ 10	≥ 40	≤ 10	9.3 (5.5, 15.7)	8.6 (2.5)	–	139 (63)	82 (37)
Salt^b^	mg	< 2000	≥ 2000/1000 kcal	≤ 1000/1000 kcal	1194 (844, 1802)	9.2 (1.8)	–	182 (82)	39 (18)
Fats and oils^b^	%energy	< 25	≥ 50	≤ 25	20 (16, 24)	9.8 (0.7)	–	180 (81)	41 (19)
Water^a^	mL	3000	0	3000	1291 (985, 1751)	4.7 (2)	215 (97)	6 (3)	–
Limit coffee^b^	Cup	0, max 3	≥ 6	0	0 (0, 0)	9.8 (0.5)	–	221 (100)	0 (0)
Have breakfast^a^	Yes/no	Daily	None (0)	All days (3)	3 (2, 3)	7.9 (2.8)	93 (42)	128 (58)	–

For each component (e.g., vegetables, fruits, protein‐rich foods), the recommended intake is shown alongside the actual median intake and interquartile range (Q1, Q3). The scoring system assigns adherence scores from 0 (non‐adherence) to 10 (full adherence), with proportional scores for intakes between the minimum and maximum standards. Moderation components, such as sugar and salt, are scored based on adherence to recommended upper limits. “Intake” represents median daily values with interquartile ranges. “Score” indicates the mean (SD) adherence score, where higher scores denote closer adherence to guidelines. “Under,” “Meeting,” and “Over” columns show the percentage of participants falling below, meeting, or exceeding recommendations. The mean total adherence score across all components was 68.5 (SD 7.3), indicating moderate overall adherence.

^a^ Adequacy component.

^b^ Moderation component.

Figure 1b highlights discrepancies between recommended and actual food group consumption according to MyPlate (Isi Piringku). While starchy staples should constitute 33% of a meal, they accounted for 68%, more than double the recommendation. Vegetable intake was particularly low at 6% instead of 33%, while fruit intake was only 4% compared to the recommended 17%. Protein‐rich food intake (22%) was closer to the suggested 17%.

### 3.2 Sociodemographic Characteristics and Association With Adherence

Participants were predominantly aged 20–35, with equal urban and rural distribution (Table 2). Over half had ≤ 9 years of education, and fewer than 10% attended university. Nearly 90% were housewives, the majority exclusively breastfeeding. One‐third were first‐time mothers, and a quarter lived in households with > 6 family members.

**Table 2:** Sociodemographic Characteristics and Estimated Mean (SE) Adherence Scores to IDG among Lactating Women (n = 220).

Characteristics	*n*	%	Vegetable	Fruit	Protein‐rich food	Starchy staples	Sugar	Salt	Fats and Oils	Water	Coffee	Breakfast
**Age**												
< 20	23	10%	1.9 (0.4)	3.8 (0.9)	9.0 (0.4)	4.2 (0.6)	9.5 (0.6)	9.3 (0.4)	9.4 (0.2)	4.3 (0.5)	9.8 (0.1)	8.2 (0.7)
20–35	164	74%	1.8 (0.1)	3.6 (0.3)	8.9 (0.1)	3.8 (0.2)	8.5 (0.2)	9.2 (0.1)	**9.8 (0.1)**	4.9 (0.2)	9.8 (0.0)	7.9 (0.2)
> 35	33	16%	2.0 (0.3)	5.8 (0.7)	8.8 (0.3)	3.9 (0.5)	8.4 (0.5)	9.0 (0.3)	9.8 (0.1)	3.9 (0.4)	9.7 (0.1)	7.6 (0.5)
**Location**												
Rural	107	49%	1.7 (0.2)	3.8 (0.4)	8.7 (0.2)	3.5 (0.3)	8.7 (0.3)	9.5 (0.2)	9.8 (0.1)	5.0 (0.2)	9.8 (0.1)	8.9 (0.3)
Urban	113	51%	1.9 (0.2)	4.1 (0.4)	9.1 (0.2)	**4.3 (0.3)**	8.5 (0.3)	**8.9 (0.2)**	9.7 (0.1)	4.5 (0.2)	9.7 (0.1)	**7.0 (0.3)**
**Education**												
Elementary School	43	20%	1.9 (0.3)	3.8 (0.6)	8.3 (0.3)	3.3 (0.4)	8.6 (0.4)	9.0 (0.3)	9.8 (0.1)	4.7 (0.3)	9.9 (0.1)	7.7 (0.5)
Junior High School	76	35%	1.7 (0.2)	4.1 (0.4)	8.9 (0.2)	3.8 (0.3)	8.3 (0.3)	9.1 (0.2)	9.7 (0.1)	4.2 (0.2)	9.7 (0.1)	7.6 (0.3)
Senior High School	85	39%	1.8 (0.2)	3.8 (0.4)	9.2 (0.2)	4.0 (0.3)	8.9 (0.3)	9.3 (0.2)	9.7 (0.1)	5.0 (0.2)	9.7 (0.1)	8.1 (0.3)
University	16	7%	2.2 (0.4)	4.1 (1.0)	9.0 (0.5)	**5.0 (0.7)**	8.4 (0.7)	9.5 (0.5)	9.8 (0.2)	5.7 (0.5)	9.9 (0.1)	9.0 (0.7)
**Occupation**												
Housewife	196	89%	1.8 (0.1)	4.0 (0.3)	9.0 (0.1)	3.9 (0.2)	8.6 (0.2)	9.2 (0.1)	9.8 (0.1)	4.7 (0.1)	9.8 (0.0)	7.9 (0.2)
Other	24	11%	1.9 (0.3)	3.6 (0.8)	8.4 (0.4)	4.0 (0.5)	8.6 (0.5)	9.4 (0.4)	9.7 (0.2)	4.8 (0.4)	9.9 (0.1)	7.8 (0.6)
**Wealth index**												
Lowest	44	20%	1.8 (0.2)	3.6 (0.6)	8.7 (0.3)	2.9 (0.4)	8.9 (0.4)	9.1 (0.3)	9.8 (0.1)	4.3 (0.3)	9.7 (0.1)	8.0 (0.4)
Second	46	21%	1.4 (0.2)	4.5 (0.6)	9.3 (0.3)	**4.3 (0.4)**	8.6 (0.4)	9.2 (0.3)	9.8 (0.1)	4.5 (0.3)	9.8 (0.1)	7.7 (0.4)
Middle	45	20%	2.0 (0.2)	3.9 (0.5)	8.7 (0.3)	3.8 (0.4)	8.4 (0.4)	9.3 (0.3)	9.8 (0.1)	4.8 (0.3)	9.8 (0.1)	7.8 (0.4)
Fourth	44	20%	2.1 (0.2)	4.0 (0.6)	9.0 (0.3)	4.0 (0.4)	9.0 (0.4)	9.2 (0.3)	9.7 (0.1)	4.8 (0.3)	9.9 (0.1)	7.7 (0.4)
Highest	41	19%	1.8 (0.3)	3.7 (0.6)	8.7 (0.3)	**4.3 (0.4)**	8.0 (0.4)	9.1 (0.3)	9.8 (0.1)	5.2 (0.3)	9.8 (0.1)	8.4 (0.5)
**Breastfeeding practice**												
Partial breastfeeding	38	17%	1.7 (0.3)	3.8 (0.6)	8.8 (0.3)	3.7 (0.4)	9.1 (0.4)	9.3 (0.3)	9.6 (0.1)	4.9 (0.3)	9.8 (0.1)	7.3 (0.5)
Exclusive breastfeeding	182	83%	1.8 (0.1)	4.0 (0.3)	8.9 (0.1)	3.9 (0.2)	8.5 (0.2)	9.1 (0.1)	9.8 (0.1)	4.7 (0.1)	9.8 (0.0)	8.0 (0.2)
**Parity**												
Primiparous	72	33%	1.6 (0.2)	4.3 (0.5)	8.8 (0.2)	3.9 (0.3)	8.6 (0.4)	9.1 (0.3)	9.8 (0.1)	4.9 (0.3)	9.9 (0.1)	7.6 (0.4)
Multiparous	148	67%	1.9 (0.1)	3.8 (0.3)	9.0 (0.2)	3.9 (0.2)	8.6 (0.2)	9.2 (0.2)	9.7 (0.1)	4.6 (0.2)	**9.7 (0.0)**	8.0 (0.2)
**Family size**												
3–4	86	39%	1.9 (0.2)	4.4 (0.4)	8.9 (0.2)	4.1 (0.3)	8.6 (0.3)	9.5 (0.2)	9.8 (0.1)	5.1 (0.2)	9.8 (0.1)	8.1 (0.3)
5–6	80	36%	1.8 (0.2)	3.8 (0.4)	9.0 (0.2)	3.7 (0.3)	8.7 (0.3)	8.9 (0.2)	9.8 (0.1)	4.6 (0.2)	9.7 (0.1)	8.1 (0.3)
> 7	54	25%	1.8 (0.2)	3.6 (0.5)	8.7 (0.2)	3.8 (0.3)	8.5 (0.3)	9.1 (0.3)	9.7 (0.1)	**4.3 (0.3)**	9.8 (0.1)	7.2 (0.4)

Bold values indicate statistical significance at *p* < 0.05. The analysis was conducted using multiple linear regression, with adherence scores as a continuous outcome variable and sociodemographic characteristics as categorical variables. The first category of each sociodemographic variable was used as the reference level for comparisons. Estimated means from the regression model are presented and mean differences (95% CI) with *p* values are available in Supplementary Table 1a–b. Trend analysis for ordinal variables found significant associations between education level and adherence to starchy staples (*p* < 0.001), wealth index and adherence to starchy staples (*p* = 0.002) and water intake (*p* = 0.041), as well as family size with water intake (*p* = 0.034) and having breakfast (*p* = 0.008).

Vegetable and fruit intake was low (0.5 and 1 serving/day, respectively) with no sociodemographic associations. Protein intake was high (3.5 servings/day) across all sociodemographic groups. Starchy staple consumption (7.1 servings/day) exceeded recommendations, with higher adherence among urban, educated, and wealthier participants.

Sugar intake contributed 9.3% of total energy, aligning with recommendations. Median sodium intake was 1200 mg/day, with 18% exceeding the 2000 mg limit, and lower adherence in urban participants. Fats and oils contributed 20% of energy, meeting recommendations.

Adherence to coffee consumption was high, though lower among multiparous women. Median water intake (1300 mL/day) was well below the 3000 mL recommendation, with lower adherence in lower‐income and larger‐family households. More than half consumed breakfast daily, but adherence was lower in urban and larger‐family households.

### 3.3 Association of Adherence to IDG With Micronutrient Adequacy

Adherence to dietary guidelines demonstrated varying relationships with nutrient adequacy across food components, as measured by Probability of Adequacy (PA) scores for 11 micronutrients (Table 3).

**Table 3:** Correlation coefficients (95% CI) between adherence scores to IDG and nutrient adequacy (probability of adequacy scores).

Adherence Component	Correlation (95% CI) with nutrient adequacy (as PA score)											
	Total PA Score	Vitamin A (RAE)	Thiamine	Riboflavin	Niacin	Vitamin B6	Folate	Vitamin B12	Vitamin C	Iron	Zinc	Calcium
Vegetables^a^	−0.10 (−0.23 −0.04)	**0.19 (0.06 −0.32)**	**−0.16 (−0.29 ‐ −0.03)**	−0.07 (−0.20 −0.06)	−0.07 (−0.20 −0.07)	−0.08 (−0.21 −0.06)	−0.06 (−0.19 −0.07)	−0.10 (−0.23 −0.03)	0.00 (−0.13 −0.14)	**−0.14 (−0.26 −0.00)**	−0.12 (−0.25 −0.01)	−0.00 (−0.13 −0.13)
Fruits^a^	0.10 (−0.03 −0.23)	**0.17 (0.03 −0.29)**	−0.01 (−0.15 −0.12)	0.07 (−0.06 −0.20)	0.00 (−0.13 −0.13)	0.03 (−0.10 −0.16)	0.10 (−0.03 −0.23)	−0.02 (−0.15 −0.11)	**0.21 (0.08 −0.33)**	0.00 (−0.13 −0.13)	**0.14 (0.01 −0.27)**	0.03 (−0.10 −0.16)
Protein foods^a^	**0.19 (0.06 −0.32)**	0.04 (−0.09 −0.17)	0.04 (−0.09 −0.17)	0.09 (−0.04 −0.22)	**0.27 (0.14 −0.39)**	0.08 (−0.05 −0.21)	0.01 (−0.12 −0.14)	**0.23 (0.11 −0.35)**	0.02 (−0.12 −0.15)	**0.20 (0.07 −0.32)**	0.03 (−0.11 −0.16)	0.10 (−0.03 −0.23)
Starchy staples^b^	0.09 (−0.05 −0.22)	0.08 (−0.06 −0.21)	0.05 (−0.08 −0.18)	−0.02 (−0.15 −0.12)	0.13 (−0.00 −0.26)	0.12 (−0.01 −0.25)	−0.03 (−0.16 −0.11)	0.02 (−0.11 −0.15)	0.12 (−0.01 −0.25)	0.03 (−0.10 −0.16)	0.04 (−0.09 −0.17)	0.05 (−0.08 −0.18)
Sugar^b^	0.02 (−0.11 −0.15)	−0.06 (−0.19 −0.08)	0.01 (−0.12 −0.14)	0.06 (−0.07 −0.19)	0.04 (−0.09 −0.17)	−0.00 (−0.13 −0.13)	0.03 (−0.10 −0.16)	−0.02 (−0.15 −0.12)	−0.06 (−0.19 −0.07)	0.08 (−0.05 −0.21)	−0.01 (−0.14 −0.12)	0.04 (−0.10 −0.17)
Salt^b^	0.03 (−0.10 −0.16)	0.10 (−0.04 −0.23)	−0.10 (−0.23 −0.04)	0.03 (−0.10 −0.16)	0.06 (−0.07 −0.20)	−0.01 (−0.14 −0.12)	0.03 (−0.10 −0.16)	−0.03 (−0.16 −0.11)	0.02 (−0.12 −0.15)	0.01 (−0.12 −0.14)	0.08 (−0.05 −0.21)	−0.01 (−0.14 −0.13)
Fats and oils^b^	0.07 (−0.07 −0.20)	0.01 (−0.12 −0.14)	0.09 (−0.05 −0.22)	0.02 (−0.11 −0.15)	0.05 (−0.08 −0.18)	−0.10 (−0.23 −0.03)	0.12 (−0.02 −0.24)	−0.03 (−0.16 −0.10)	0.02 (−0.11 −0.16)	0.06 (−0.07 −0.19)	0.12 (−0.01 −0.25)	0.05 (−0.08 −0.18)
Water^a^	**0.26 (0.13 −0.38)**	**0.19 (0.06 −0.31)**	0.07 (−0.07 −0.20)	**0.20 (0.07 −0.33)**	**0.25 (0.12 −0.37)**	**0.13 (0.00 −0.26)**	0.11 (−0.02 −0.24)	**0.16 (0.03 −0.29)**	0.05 (−0.08 −0.18)	**0.16 (0.03 −0.28)**	0.10 (−0.03 −0.23)	**0.17 (0.04 −0.29)**
Limit coffee^b^	0.01 (−0.13 −0.14)	0.00 (−0.13 −0.13)	−0.02 (−0.15 −0.12)	−0.02 (−0.15 −0.11)	0.02 (−0.11 −0.15)	0.10 (−0.03 −0.23)	−0.11 (−0.24 −0.02)	0.03 (−0.10 −0.16)	0.00 (−0.13 −0.14)	−0.02 (−0.15 −0.12)	0.05 (−0.08 −0.18)	0.06 (−0.08 −0.19)
Have breakfast^a^	**0.22 (0.09 −0.34)**	**0.16 (0.03 −0.29)**	**0.14 (0.00 −0.26)**	**0.16 (0.03 −0.29)**	**0.17 (0.04 −0.30)**	0.06 (−0.07 −0.19)	0.08 (−0.05 −0.21)	0.09 (−0.05 −0.22)	0.12 (−0.01 −0.25)	**0.20 (0.07 −0.33)**	0.07 (−0.07 −0.20)	0.06 (−0.08 −0.19)

Bold values indicate statistical significance at *p* < 0.05. Nutrient adequacy is represented as the Probability of Adequacy (PA) score, calculated based on the Estimated Average Requirement (EAR). Data are presented as Pearson correlation coefficients (*r*) with 95% confidence intervals (CI). The Pearson correlation coefficient ranges from −1 to 1, where positive values indicate a direct association between adherence scores and nutrient adequacy, while negative values indicate an inverse relationship. Nutrient intake and PA for each nutrient are available in Supplementary Table 2.

^a^ Adequacy component.

^b^ Moderation component.

Protein‐rich food adherence correlated positively with niacin, vitamin B12, iron, and overall score (*r* = 0.19–0.27). Drinking water adherence showed associations with multiple nutrients, including vitamin A, riboflavin, niacin, vitamin B12, iron, calcium, and overall score (*r* = 0.16–0.25). Breakfast adherence correlated with niacin, iron, and riboflavin (*r* = 0.16–0.20). Fruit adherence was positively associated with vitamins A, C, and zinc (*r* = 0.14–0.21).

Vegetable adherence correlated positively with vitamin A (*r* = 0.19, 95% CI: 0.06, 0.32) but negatively with thiamine and iron (*r* = −0.16, −0.14). No significant correlations were observed between adherence to starchy staples intake and limiting sugar, salt, and fats.

### 3.4 Nutritional Biomarkers Associated With Adherence

Associations of adherence to dietary guidelines and maternal nutritional biomarkers, infant growth, or micronutrient status are available in Table 4. Fruit adherence correlated positively with maternal B12 (*β* = 6.07, 95% CI: 1.76, 10.38) but negatively with infant ferritin (*β* = −1.85, 95% CI: −3.45, −0.24). Moderate starchy staple intake was linked to higher maternal zinc (*β* = 0.12, 95% CI: 0.01, 0.22).

**Table 4:** Heatmap of beta coefficients from multivariate regression between adherence scores and nutrient biomarkers in mothers and infants.

Component	Mother										Infant						
	Obesity	Blood marker				Breastmilk					Growth			Blood marker			
	BMI	Iron	Zinc	Vit A	Vit B12	Volume	Iron	Zinc	Vit A	Vit B12	WAZ	LAZ	WLZ	Iron	Zinc	Vit A	Vit B12
	(kg/m^2^)	(μg/L)	(µmol/L)	(μg/L)	(pmol/L)	(mL/d)	(μg/L)	(μg/L)	(μg/L)	(pmol/L)	*z*‐score	*z*‐score	*z*‐score	(μg/L)	(µmol/L)	(μg/L)	(pmol/L)
Mean	24.9	35.9	10.4	1.4	298.4	680	0.2	0.9	0.4	242.1	−0.60	−0.69	0.08	46.5	11.3	1.0	258.5
SD	3.7	29.7	1.3	0.4	115.2	219	0.2	0.5	0.2	154.6	0.86	0.96	1.01	35.6	2.0	0.2	138.4
Vegetables^a^	0.25	0.84	−0.01	0.04	−4.98	0.04	0.00	−0.04	0.02	−2.32	0.03	0.03	0.06	2.82	0.01	0.00	−2.41
Fruits^a^	0.00	0.56	−0.03	0.00	**6.07**	1.12	0.00	0.00	0.01	3.69	0.00	0.00	−0.04	**−1.85**	−0.07	0.00	2.38
Protein foods^a^	−0.06	1.20	−0.01	0.01	2.09	13.33	0.01	0.00	0.00	1.08	0.01	0.08	0.08	−3.92	−0.06	0.01	−0.64
Starchy staples^b^	−0.15	−1.45	**0.12**	−0.01	−2.51	−3.83	−0.01	0.00	0.01	−7.60	−0.03	−0.02	−0.09	1.79	0.09	−0.01	−1.65
Sugar^b^	−0.04	**−2.90**	0.10	0.00	−3.39	−1.88	−0.01	−0.02	0.01	−0.45	0.02	−0.04	−0.02	1.95	0.13	−0.01	0.55
Salt^b^	0.15	1.15	**−0.11**	−0.03	−0.58	10.70	0.00	0.00	−0.01	2.73	−0.01	−0.10	0.01	−3.00	0.02	0.00	−3.65
Fats and oils^b^	−0.50	1.35	0.03	−0.04	**−23.08**	22.04	−0.02	−0.02	−0.04	**−36.71**	0.07	−0.04	0.06	−8.02	0.08	−0.01	−8.94
Water^a^	0.08	−1.02	0.05	−0.02	−0.54	−5.29	0.00	0.01	0.00	−4.65	−0.01	−0.03	0.02	1.06	−0.09	0.01	4.12
Limit coffee^b^	0.29	1.18	−0.11	0.10	1.36	**−55.73**	0.02	0.07	0.01	2.63	**−0.37**	−0.05	−0.38	4.41	−0.04	**−0.10**	34.42
Have breakfast^a^	−0.15	0.19	−0.01	0.00	5.55	7.07	0.00	0.00	0.00	7.08	−0.03	−0.04	−0.10	0.67	−0.03	0.00	0.54

*Note:* The heatmap shows the beta coefficient between adherence scores for dietary components and nutrition biomarkers for mothers and infants. Positive correlations are blue, negative are red, and bolded values are significant (*p* < 0.05). Maternal biomarkers include BMI, iron, zinc, vitamin A (RBP), and vitamin B12 levels, and breastmilk iron, zinc, vitamin A, and vitamin B12. Infant outcomes include WAZ, HAZ, WHZ, and nutrient biomarkers (iron, zinc, vitamin A, and vitamin B12). Adherence scores (1–10) were analysed using multivariate regression without standardising effect sizes. Covariates for the analysis included maternal age, total energy intake, and nutrient‐specific intake (e.g., dietary iron for serum ferritin) for maternal blood biomarkers; milk volume, exclusive breastfeeding status, and nutrient‐specific intake for breastmilk composition; exclusive breastfeeding, birth size (length and weight), infant inflammation markers, infant energy intake for infant growth indicators; and maternal/infant nutrient‐specific intake for infant blood biomarkers. Nutritional status biomarker values were adjusted for inflammation markers. Detailed 95% confidence intervals (CIs) for these correlations are provided in Supplementary Table 3a–c.

^a^ Adequacy component.

^b^ Moderation component.

Negative associations were observed for limiting certain food components. Restricting salt was linked to lower maternal zinc (*β* = −0.11, 95% CI: −0.22, −0.00). Limiting fats and oils correlated with reduced maternal B12 (*β* = −23.08, 95% CI: −45.70, −0.45) and breastmilk B12 (*β* = −36.71, 95% CI: −69.13, −4.29). Coffee restriction was associated with reduced breastmilk volume (*β* = −55.7, 95% CI: −106.7, −4.7), lower infant WAZ (*β* = −0.37, 95% CI: −0.65, −0.10), and lower infant RBP (*β* = −0.10, 95% CI: −0.17, −0.03). Sugar restriction was negatively associated with maternal iron (*β* = −2.90, 95% CI: −5.55, −0.25).

No significant associations were found for adherence to vegetable, protein‐rich food, water intake, or breakfast with any biomarkers.

## 4 Discussion

This study found low adherence to IDG among lactating women, particularly for vegetables, fruits, and starchy staples, with rice intake often exceeding recommendations, while protein intake was relatively better. Participants followed other moderation‐based guidelines (sugar, salt, fat) more than consumption recommendations for primary food groups.

Our analysis highlights that while adherence to dietary guidelines aligned with nutrient intake and adequacy, it did not consistently translate into improved nutrient biomarkers. This discrepancy suggests that focusing solely on nutrient intake adequacy may not be sufficient to improve nutritional biomarkers. An important consideration is the potential mismatch between existing reference values for nutrient adequacy during lactation, and the actual physiological requirements of lactating women (Allen et al. 2018). This highlights the need for updated, lactation‐specific reference values and refined food‐based dietary guidelines that better address key health challenges, particularly in Indonesia, where micronutrient deficiencies, child growth failure, and obesity remain pressing concerns.

Given limited research on dietary adherence and nutritional biomarkers in lactating women (Rahmannia et al. 2025), this study explored associations between dietary adherence and nutritional status indicators without a predefined hypothesis. While no consistent patterns emerged, some dietary components showed notable associations with specific biomarkers, highlighting areas for dietary guideline refinement. As this was an exploratory study, multiple testing corrections were not applied to enable a comprehensive assessment across all dietary components and biomarkers. While such corrections can reduce the risk of Type I errors, they could also increase Type II error risk, potentially obscuring meaningful associations and limiting the identification of key dietary factors for future research. For instance, the observed negative association between coffee restriction and infant WAZ may reflect reverse causality or unmeasured confounding, warranting further investigation.

### 4.1 High Starchy Staple Consumption and Its Impact on Maternal Nutrition

Starchy staple intake, predominantly white rice, far exceeded recommendations (Ministry of Health Republic Indonesia 2014). As a cultural and economic staple for over 90% of Indonesians (Khairunnisa et al. 2022; Rusdi 2023; World Food Programme, & Kementerian PPN/Bappenas RI 2017), white rice often dominates meals, with protein and vegetables in small amounts (Rahmannia et al. 2019). Adherence to recommendations (i.e., moderate intake) was lower among participants with less education, rural residency, and lower wealth, likely reflecting limited access to diverse foods and nutrition knowledge.

Our study found no positive correlation between adherence to starchy staple recommendations, meaning consumption in moderation rather than excess, and micronutrient adequacy. This suggests that simply meeting recommended intake levels does not inherently improve micronutrient adequacy unless accompanied by a more diverse and nutrient‐rich diet. Moreover, exceeding the recommended intake for starchy staples was linked to lower maternal zinc status, likely due to the limited zinc content in refined grains. High white rice consumption may also reduce dietary diversity by displacing vegetables, fruits, and protein‐rich foods. This aligns with findings that excessive reliance on rice as a starchy staple is associated with micronutrient deficiencies in populations with limited dietary diversity (Piccoli et al. 2012). Interestingly, while high starchy staple intake was associated with lower maternal zinc levels, it did not significantly affect iron status. This may be because lactation suppresses menstruation, reducing maternal iron loss and preserving iron stores (Institute of Medicine IOM 2001).

Low adherence to grain recommendations among lactating women has also been reported in Australia and the United States, where refined grain intake is high (Morrison et al. 2012; Nansel et al. 2022). However, staple grain intake in our study population exceeded levels observed elsewhere (Ferranti et al. 2019; Jardí et al. 2019; Slater et al. 2020; von Ruesten et al. 2014). This trend may reflect limited awareness of serving sizes, leading to double portions, and economic constraints prioritising low‐cost, high‐energy foods like white rice over nutrient‐dense options. In lower‐income households, affordability and food security concerns may reinforce high reliance on starchy staple foods. These findings highlight the need for clearer portion guidance in dietary guidelines and education on serving sizes, alongside strategies to improve access to affordable, nutrient‐dense alternatives.

### 4.2 Low Vegetable and Fruit Intake Among Lactating Women

Few lactating women met recommended vegetable and fruit intake levels, highlighting a significant dietary gap. Adherence to vegetable recommendations correlated positively with vitamin A adequacy but showed no association with other micronutrients, nutrient biomarkers, BMI, or infant growth. Negative correlations with several micronutrients suggest that higher vegetable intake did not consistently improve overall nutrient adequacy.

Adherence to fruit recommendations correlated positively with maternal vitamin A and vitamin C intake adequacy, and serum B12 but was unexpectedly linked to lower infant iron status, while maternal iron status showed a positive trend. This may reflect infants' reliance on prenatal iron stores, sustaining them through the first 6 months (Baker et al. 2010; Sharon et al. 2019). The positive correlation between fruit intake and maternal serum B12 levels is likely an indirect effect rather than a direct dietary source of B12, as fruits do not naturally contain this vitamin. A plausible explanation is that the acid content in fruits might aid in the release and absorption of B12, though further research is needed to confirm this effect (Schubert 2014).

Vegetable intake did not vary by wealth or family size, suggesting cultural and behavioural rather than economic factors influence consumption. Despite access to fresh produce, rural residents had lower adherence than urban counterparts, possibly prioritising sales over personal consumption (Abdoellah et al. 2020). Social and cultural norms also shape dietary choices, often relegating vegetables behind starchy staples like rice (Monterrosa et al. 2020). In contrast, fruit intake was more affected by economic factors, with larger families consuming less, likely due to cost.

Low fruit and vegetable intake is common in Indonesia and globally, particularly among women (Darmawan et al. 2023; Ferranti et al. 2019; Filatava et al. 2024). This raises questions about the feasibility of current dietary recommendations, as many individuals struggle to meet the prescribed intake. For example, the guideline recommends 3–4 servings of vegetables and 2–3 servings of fruit daily, totalling 5–7 servings, yet a meta‐analysis suggests health benefits plateau at five servings, with no further mortality reduction beyond this (Toh et al. 2020; Wang et al. 2014). This suggests that setting more achievable intake targets could still provide substantial health benefits while being more culturally realistic for populations facing dietary barriers.

### 4.3 Adequate Protein‐Rich Food Intake and the Need for Separate Animal versus Plant Protein Guidance

Overall protein‐rich food intake among participants aligned with IDG recommendations. While adherence correlated positively with nutrient adequacy, no significant associations were found between protein intake and nutritional biomarkers. This may be due to assessing total protein intake rather than differentiating between animal and plant sources, which have distinct nutrient profiles and bioavailability.

Animal‐based protein foods, such as meat, fish, and eggs, generally provide more bioavailable essential nutrients like zinc, iron, and vitamin B12 compared to plant‐based sources (Hurrell and Egli 2010; McAfee et al. 2010). This is particularly relevant for lactating women, whose higher nutrient demands support maternal health and milk production (Allen et al. 2018). While research on protein source differentiation in lactation is limited, one study found that breastmilk vitamin A composition was positively associated with vitamin A intake from animal products rather than plant sources (Nimmannun et al. 2022).

Over the past 2 years, the Indonesian Ministry of Health has started campaigns to promote animal protein consumption during National Nutrition Day, encouraging the inclusion of animal and plant proteins in daily meals (Ministry of Health Republic Indonesia 2023). However, the current IDG does not differentiate between plant‐ and animal‐based protein recommendations despite their distinct nutritional profiles. Given the role of animal proteins in providing bioavailable nutrients critical for lactation, further research is needed to assess whether explicit dietary recommendations for animal versus plant protein could better support maternal and infant health.

Protein adherence did not differ by sociodemographic factors, suggesting it is widely accepted among lactating women. This broad acceptance presents an opportunity for targeted nutritional interventions, building upon an existing foundation of adherence to support maternal and infant health.

### 4.4 Re‐Evaluating Water Intake Guidelines for Lactating Women

The IDG recommendation of 3000 mL/day of drinking water for lactating women may be unrealistic, as the median intake in our study was only 1300 mL/day. Over half relied on bottled water, suggesting affordability constraints, especially for low‐income and larger families. Research shows that only half of total water intake comes from drinking water, with the rest obtained from food sources (Zhou et al. 2019), aligning with our findings. Further research is needed to explore cultural perceptions and household prioritisation of water consumption.

Unlike the IDG, international guidelines recommend total fluid intake rather than just drinking water. The European Food Safety Authority (EFSA) suggests 2700 mL/day, including fluids from food (EFSA Panel on Dietetic Products, N., and Allergies NDA 2010), implying a lower drinking water requirement.

While hydration is essential, increasing water intake beyond physiological needs may not provide additional benefits for breastfeeding outcomes. Higher water intake was not associated with increased breastmilk volume, consistent with previous research and systematic reviews showing that extra fluid does not enhance milk production (McKenzie et al. 2015; Ndikom et al. 2014). In addition, generalised water intake recommendations may overlook individual variation in hydration needs and fail to account for fluid contributions from high‐water‐content foods such as fruits, vegetables, and soups (Rosinger 2020). During lactation, the body adapts to increased fluid output by conserving water via reduced urinary losses, and most women regulate their fluid intake based on physiological cues like thirst (McKenzie et al. 2017). Practical, evidence‐based hydration strategies, such as encouraging drinking to thirst and integrating water‐rich foods into daily meals, may be more realistic and culturally appropriate than fixed volume targets.

### 4.5 Strengths and Limitations

This is the first study in LMICs to assess adherence to food‐based dietary guidelines among lactating women and its relationship with nutritional biomarkers, addressing a key research gap. The study provides a comprehensive evaluation of adherence, influencing factors, nutrient adequacy, and outcomes, with participants from urban and rural areas to enhance representativeness.

However, the focus on West Java may limit the generalisability of the findings to other regions of Indonesia, given the country's diverse cultures and dietary habits. Nevertheless, for certain food group intakes, we found the results comparable with those from other areas in Indonesia. While adequate for detecting differences among larger sociodemographic groups, the sample size is relatively small for subgroup analyses. Additionally, specific components, such as adherence score to coffee consumption guidelines, exhibited minimal variability among participants, which could reduce the study's capacity to explore associations with other factors or outcomes.

Despite adjustments for inflammation, time of day, and fasting status, serum zinc remains sensitive to physiological variation and tends to respond more to supplementation than food‐based intake, potentially explaining modest associations observed (King et al. 2016). Nonetheless, it remains the best available biomarker of population‐level zinc status (de Benoist et al. 2007). Serum vitamin B12 may fail to detect early deficiency, as total concentrations can remain normal despite tissue depletion. The absence of more sensitive markers, such as holotranscobalamin (holoTC) or serum methylmalonic acid (MMA), may have limited the interpretation of B12‐related findings (Allen et al. 2018).

As an exploratory study, we used Pearson correlations and linear regression models to examine a broad set of associations. While this approach was suitable for identifying general patterns, it assumes linear and additive relationships and does not capture potential nonlinear or moderated effects. Future hypothesis‐driven research should consider exploring interaction terms and nonlinear dose–response patterns using continuous intake data where feasible. We also did not apply multiple testing corrections to avoid inflating Type II errors in this exploratory context. However, this increases the risk of false positives, and findings should be interpreted as preliminary signals warranting further investigation.

## 5 Conclusions

This study highlights moderate adherence to the IDG among lactating women, with low adherence to starchy staple, vegetable, and fruit recommendations, while guidelines for limiting sugar, salt, and fat were better followed. The disconnect between adherence, nutrient intake adequacy, and outcomes suggests the need to refine dietary guidelines to better support lactating women's needs, particularly in addressing micronutrient deficiencies and child growth.

Policy recommendations from this study highlight areas where refinements to dietary guidelines and public health interventions may better support maternal and child nutrition. First, clearer portion guidance on starchy staples could help promote dietary variety and balanced rice consumption, ensuring that staple intake does not displace other nutrient‐rich foods. Differentiating protein recommendations for animal‐ and plant‐based sources may be warranted to ensure that the higher bioavailability of key micronutrients in animal‐source protein foods is recognised and optimised within dietary guidance.

Given the benefits of fruit consumption but its low adherence among participants and considering that no sociodemographic factors were significantly associated with low fruit intake, a multifaceted approach may be necessary. Targeted education and behaviour change programmes could play a role in promoting fruit consumption by addressing potential cultural and behavioural barriers. At the same time, policies aimed at improving affordability and availability, particularly in lower‐income communities, may help increase access to fresh produce. Finally, adjusting the 3000 mL/day drinking water guideline to better align with international total fluid intake recommendations could improve feasibility and adherence.

## Author Contributions

Sofa Rahmannia, Kevin Murray, Gina Arena, Rosalind Gibson and Siobhan Hickling designed the study. Sofa Rahmannia and Aly Diana performed the investigation. Sofa Rahmannia and Kevin Murray analysed the data and visualisation. Sofa Rahmannia wrote the manuscript. Gina Arena, Kevin Murray, Siobhan Hickling, Aly Diana and Rosalind Gibson reviewed and edited the manuscript. All authors have read and approved the final manuscript.

## Conflicts of Interest

The authors declare no conflicts of interest.

## Supporting information


**Supporting Table 1a:** Detailed Statistical Analysis of Sociodemographic Factors Associated with Adherence to Each Dietary Guideline Message. **Supporting Table 1b:** Detailed Statistical Analysis of Sociodemographic Factors Associated with Adherence to Each Dietary Guideline Message (cont'd). **Supporting Table 2:** Summary of Nutrient Intake and Probability of Adequacy (PA) Based on Estimated Average Requirements (EAR). **Supporting Table 3a:** Beta Coefficients (95% CI) Between Adherence Scores and Nutrition Biomarker: Mother Nutrition Biomarker. **Supporting Table 3b:** Beta Coefficients (95% CI) Between Adherence Scores and Nutrition Biomarker: Breastmilk Composition. **Supporting Table 3c:** Beta Coefficients (95% CI) Between Adherence Scores and Nutrition Biomarker: Infant Nutrition Biomarker.

## Data Availability

The data that support the findings of this study are available from the corresponding author upon reasonable request.
