# Supplementary material for: Adherence to Indonesia's Dietary Guidelines Among Lactating Women: Insights for Policy and Practice
Source: Matern Child Nutr. 2025 Jul 29;21(4):e70075. doi: 10.1111/mcn.70075 (PMC12454190; doi:10.1111/mcn.70075)
Supplement: Supplementary file 1 — Supporting Table 1a: Detailed Statistical Analysis of Sociodemographic Factors Associated with Adherence to Each Dietary Guideline Message. Supporting Table 1b: Detailed Statistical Analysis of Sociodemographic Factors Associated with Adherence to Each Dietary Guideline Message (cont'd). Supporting Table 2: Summary of Nutrient Intake and Probability of Adequacy (PA) Based on Estimated Average Requirements (EAR). Supporting Table 3a: Beta Coefficients (95% CI) Between Adherence Scores and Nutrition Biomarker: Mother Nutrition Biomarker. Supporting Table 3b: Beta Coefficients (95% CI) Between Adherence Scores and Nutrition Biomarker: Breastmilk Composition. Supporting Table 3c: Beta Coefficients (95% CI) Between Adherence Scores and Nutrition Biomarker: Infant Nutrition Biomarker. [file MCN-21-e70075-s001.docx]

Supplementary Table 1a. Detailed Statistical Analysis of Sociodemographic Factors Associated with Adherence to Each Dietary Guideline Message

| Characteristics | n | % | **Vegetable** | | | **Fruit** | | | **Protein Food** | | | **Staple food** | | | **Daily Breakfast** | | |
| --- | --- | --- | --- | --- | --- | --- | --- | --- | --- | --- | --- | --- | --- | --- | --- | --- | --- |
|  |  |  | Est.mean (SE) | difference in means (95% CI) | p-value | Est.mean (SE) | difference in means (95% CI) | p-value | Est.mean (SE) | difference in means (95% CI) | p-value | Est.mean (SE) | difference in means (95% CI) | p-value | Est.mean (SE) | difference in means (95% CI) | p-value |
|  |  |  | 1.8 (0.1) |  |  | 4 (0.2) |  |  | 8.9 (0.1) |  |  | 3.9 (0.2) |  |  | 7.9 (0.2) |  |  |
| **Age** |  |  |  |  |  |  |  |  |  |  |  |  |  |  |  |  |  |
| <20 | 23 | 10% | 1.9 (0.4) | ref |  | 3.8 (0.9) | ref |  | 9 (0.4) | ref |  | 4.2 (0.6) | ref |  | 8.2 (0.7) | ref |  |
| 20 - 35 | 164 | 74% | 1.8 (0.1) | -0.1 (-0.9, 0.7) | 0.822 | 3.6 (0.3) | -0.2 (-2.1, 1.7) | 0.841 | 8.9 (0.1) | -0.1 (-1, 0.9) | 0.888 | 3.8 (0.2) | -0.4 (-1.7, 0.8) | 0.509 | 7.9 (0.2) | -0.2 (-1.6, 1.2) | 0.754 |
| >35 | 33 | 16% | 2 (0.3) | 0.2 (-0.8, 1.2) | 0.709 | 5.8 (0.7) | 2 (-0.3, 4.3) | 0.092 | 8.8 (0.3) | -0.2 (-1.3, 1) | 0.774 | 3.9 (0.5) | -0.3 (-1.9, 1.3) | 0.714 | 7.6 (0.5) | -0.5 (-2.3, 1.2) | 0.563 |
| **Location** |  |  |  |  |  |  |  |  |  |  |  |  |  |  |  |  |  |
| Rural | 107 | 49% | 1.7 (0.2) | ref |  | 3.8 (0.4) | ref |  | 8.7 (0.2) | ref |  | 3.5 (0.3) | ref |  | 8.9 (0.3) | ref |  |
| Urban | 113 | 51% | 1.9 (0.2) | 0.2 (-0.3, 0.7) | 0.417 | 4.1 (0.4) | 0.3 (-0.8, 1.5) | 0.560 | 9.1 (0.2) | 0.4 (-0.1, 1) | 0.150 | **4.3 (0.3)** | **0.8 (0, 1.6)** | **0.039** | **7 (0.3)** | **-1.9 (-2.8, -1.1)** | **0.000** |
| **Education** |  |  |  |  |  |  |  |  |  |  |  |  |  |  |  |  |  |
| Elementary School | 43 | 20% | 1.9 (0.3) | ref |  | 3.8 (0.6) | ref |  | 8.3 (0.3) | ref |  | 3.3 (0.4) | ref |  | 7.7 (0.5) | ref |  |
| Junior High School | 76 | 35% | 1.7 (0.2) | -0.2 (-0.8, 0.4) | 0.524 | 4.1 (0.4) | 0.4 (-1.1, 1.8) | 0.619 | 8.9 (0.2) | 0.6 (-0.1, 1.4) | 0.078 | 3.8 (0.3) | 0.5 (-0.5, 1.5) | 0.326 | 7.6 (0.3) | -0.1 (-1.1, 1) | 0.917 |
| Senior High School | 85 | 39% | 1.8 (0.2) | -0.1 (-0.7, 0.6) | 0.827 | 3.8 (0.4) | 0.1 (-1.5, 1.6) | 0.946 | 9.2 (0.2) | 0.9 (0.1, 1.6) | 0.082 | 4 (0.3) | 0.7 (-0.4, 1.8) | 0.210 | 8.1 (0.3) | 0.4 (-0.7, 1.6) | 0.468 |
| University | 16 | 7% | 2.2 (0.4) | 0.3 (-0.7, 1.3) | 0.546 | 4.1 (1) | 0.3 (-2.1, 2.7) | 0.823 | 9 (0.5) | 0.7 (-0.5, 1.9) | 0.265 | **5 (0.7)** | **1.6 (0, 3.3)** | **0.049** | 9 (0.7) | 1.3 (-0.5, 3.2) | 0.143 |
| **Occupation** |  |  |  |  |  |  |  |  |  |  |  |  |  |  |  |  |  |
| Housewife | 196 | 89% | 1.8 (0.1) | ref |  | 4 (0.3) | ref |  | 9 (0.1) | ref |  | 3.9 (0.2) | ref |  | 7.9 (0.2) | ref |  |
| Other | 24 | 11% | 1.9 (0.3) | 0.1 (-0.6, 0.8) | 0.742 | 3.6 (0.8) | -0.4 (-2, 1.2) | 0.636 | 8.4 (0.4) | -0.5 (-1.3, 0.3) | 0.210 | 4 (0.5) | 0.1 (-1, 1.2) | 0.867 | 7.8 (0.6) | -0.2 (-1.4, 1.1) | 0.793 |
| **Wealth index** |  |  |  |  |  |  |  |  |  |  |  |  |  |  |  |  |  |
| Lowest | 44 | 20% | 1.8 (0.2) | ref |  | 3.6 (0.6) | ref |  | 8.7 (0.3) | ref |  | 2.9 (0.4) | ref |  | 8 (0.4) | ref |  |
| Second | 46 | 21% | 1.4 (0.2) | -0.3 (-1, 0.3) | 0.308 | 4.5 (0.6) | 0.9 (-0.6, 2.4) | 0.255 | 9.3 (0.3) | 0.7 (-0.1, 1.4) | 0.085 | **4.3 (0.4)** | **1.4 (0.3, 2.4)** | **0.011** | 7.7 (0.4) | -0.3 (-1.5, 0.8) | 0.572 |
| Middle | 45 | 20% | 2 (0.2) | 0.2 (-0.4, 0.9) | 0.469 | 3.9 (0.5) | 0.3 (-1.3, 1.8) | 0.734 | 8.7 (0.3) | 0.1 (-0.7, 0.8) | 0.886 | 3.8 (0.4) | 0.8 (-0.2, 1.9) | 0.124 | 7.8 (0.4) | -0.2 (-1.3, 1) | 0.763 |
| Fourth | 44 | 20% | 2.1 (0.2) | 0.3 (-0.4, 1) | 0.359 | 4 (0.6) | 0.4 (-1.2, 2.1) | 0.592 | 9 (0.3) | 0.3 (-0.5, 1.1) | 0.488 | 4 (0.4) | 1.1 (0, 2.2) | 0.054 | 7.7 (0.4) | -0.4 (-1.6, 0.9) | 0.573 |
| Highest | 41 | 19% | 1.8 (0.3) | 0 (-0.8, 0.7) | 0.977 | 3.7 (0.6) | 0.1 (-1.6, 1.9) | 0.893 | 8.7 (0.3) | 0.1 (-0.8, 0.9) | 0.908 | **4.3 (0.4)** | **1.4 (0.2, 2.5)** | **0.025** | 8.4 (0.5) | 0.4 (-0.9, 1.7) | 0.572 |
| **Breastfeeding practice** | |  |  |  |  |  |  |  |  |  |  |  |  |  |  |  |  |
| Partial breastfeeding | 38 | 17% | 1.7 (0.3) | ref |  | 3.8 (0.6) | ref |  | 8.8 (0.3) | ref |  | 3.7 (0.4) | ref |  | 7.3 (0.5) | ref |  |
| Exclusive breastfeeding | 182 | 83% | 1.8 (0.1) | 0.1 (-0.4, 0.7) | 0.639 | 4 (0.3) | 0.2 (-1.1, 1.6) | 0.722 | 8.9 (0.1) | 0.1 (-0.6, 0.8) | 0.745 | 3.9 (0.2) | 0.2 (-0.8, 1.1) | 0.735 | 8 (0.2) | 0.7 (-0.3, 1.7) | 0.150 |
| **Parity** |  |  |  |  |  |  |  |  |  |  |  |  |  |  |  |  |  |
| Primiparous | 72 | 33% | 1.6 (0.2) | ref |  | 4.3 (0.5) | ref |  | 8.8 (0.2) | ref |  | 3.9 (0.3) | ref |  | 7.6 (0.4) | ref |  |
| Multiparous | 148 | 67% | 1.9 (0.1) | 0.4 (-0.2, 0.9) | 0.210 | 3.8 (0.3) | -0.6 (-1.9, 0.7) | 0.370 | 9 (0.2) | 0.2 (-0.4, 0.8) | 0.524 | 3.9 (0.2) | 0 (-0.9, 0.9) | 0.978 | 8 (0.2) | 0.4 (-0.6, 1.4) | 0.412 |
| **Family size** |  |  |  |  |  |  |  |  |  |  |  |  |  |  |  |  |  |
| 3-4 | 86 | 39% | 1.9 (0.2) | ref |  | 4.4 (0.4) | ref |  | 8.9 (0.2) | ref |  | 4.1 (0.3) | ref |  | 8.1 (0.3) | ref |  |
| 5-6 | 80 | 36% | 1.8 (0.2) | -0.1 (-0.6, 0.4) | 0.774 | 3.8 (0.4) | -0.6 (-1.8, 0.6) | 0.335 | 9 (0.2) | 0.1 (-0.5, 0.7) | 0.736 | 3.7 (0.3) | -0.4 (-1.3, 0.4) | 0.280 | 8.1 (0.3) | 0 (-0.9, 0.9) | 0.946 |
| >7 | 54 | 25% | 1.8 (0.2) | -0.1 (-0.7, 0.5) | 0.713 | 3.6 (0.5) | -0.8 (-2.1, 0.5) | 0.239 | 8.7 (0.2) | -0.2 (-0.8, 0.5) | 0.594 | 3.8 (0.3) | -0.3 (-1.2, 0.6) | 0.476 | 7.2 (0.4) | -0.9 (-1.9, 0.1) | 0.068 |

Bold values indicate statistical significance at p<0.05. Analysis was conducted using multiple linear regression (full model), with adherence scores as quantitative outcomes and sociodemographic characteristics as categorical variables

Supplementary Table 1b. Detailed Statistical Analysis of Sociodemographic Factors Associated with Adherence to Each Dietary Guideline Message (cont’d)

| Characteristics | n | % | **Limiting Sugar** | | | **Limiting Salt** | | | **Limiting Fat and Oil** | | | **Water** | | | **Coffee** | | |
| --- | --- | --- | --- | --- | --- | --- | --- | --- | --- | --- | --- | --- | --- | --- | --- | --- | --- |
|  |  |  | Est.mean (SE) | difference in means (95% CI) | p-value | Est.mean (SE) | difference in means (95% CI) | p-value | Est.mean (SE) | difference in means (95% CI) | p-value | Est.mean (SE) | difference in means (95% CI) | p-value | Est.mean (SE) | difference in means (95% CI) | p-value |
|  |  |  | 8.6 (0.2) |  |  | 9.2 (0.1) |  |  | 9.8 (0) |  |  | 4.7 (0.1) |  |  | 9.8 (0) |  |  |
| **Age** |  |  |  |  |  |  |  |  |  |  |  |  |  |  |  |  |  |
| <20 | 23 | 10% | 9.5 (0.6) | ref |  | 9.3 (0.4) | ref |  | 9.4 (0.2) | ref |  | 4.3 (0.5) | ref |  | 9.8 (0.1) | ref |  |
| 20 - 35 | 164 | 74% | 8.5 (0.2) | -1 (-2.3, 0.3) | 0.128 | 9.2 (0.1) | -0.1 (-1, 0.9) | 0.898 | **9.8 (0.1)** | **0.4 (0.1, 0.8)** | **0.025** | 4.9 (0.2) | 0.6 (-0.4, 1.6) | 0.249 | 9.8 (0) | 0 (-0.3, 0.2) | 0.785 |
| >35 | 33 | 16% | 8.4 (0.5) | -1.1 (-2.7, 0.5) | 0.192 | 9 (0.3) | -0.2 (-1.4, 0.9) | 0.683 | 9.8 (0.1) | 0.4 (-0.1, 0.9) | 0.088 | 3.9 (0.4) | -0.4 (-1.7, 0.8) | 0.487 | 9.7 (0.1) | -0.1 (-0.4, 0.2) | 0.593 |
| **Location** |  |  |  |  |  |  |  |  |  |  |  |  |  |  |  |  |  |
| Rural | 107 | 49% | 8.7 (0.3) | ref |  | 9.5 (0.2) | ref |  | 9.8 (0.1) | ref |  | 5 (0.2) | ref |  | 9.8 (0.1) | ref |  |
| Urban | 113 | 51% | 8.5 (0.3) | -0.2 (-1, 0.6) | 0.657 | **8.9 (0.2)** | **-0.6 (-1.2, -0.1)** | **0.033** | 9.7 (0.1) | -0.1 (-0.3, 0.2) | 0.587 | 4.5 (0.2) | -0.5 (-1.1, 0.1) | 0.093 | 9.7 (0.1) | -0.1 (-0.3, 0.1) | 0.203 |
| **Education** |  |  |  |  |  |  |  |  |  |  |  |  |  |  |  |  |  |
| Elementary School | 43 | 20% | 8.6 (0.4) | ref |  | 9 (0.3) | ref |  | 9.8 (0.1) | ref |  | 4.7 (0.3) | ref |  | 9.9 (0.1) | ref |  |
| Junior High School | 76 | 35% | 8.3 (0.3) | -0.3 (-1.3, 0.7) | 0.562 | 9.1 (0.2) | 0.1 (-0.6, 0.8) | 0.762 | 9.7 (0.1) | -0.1 (-0.4, 0.2) | 0.522 | 4.2 (0.2) | -0.5 (-1.3, 0.2) | 0.176 | 9.7 (0.1) | -0.2 (-0.4, 0) | 0.088 |
| Senior High School | 85 | 39% | 8.9 (0.3) | 0.2 (-0.9, 1.3) | 0.685 | 9.3 (0.2) | 0.3 (-0.5, 1) | 0.531 | 9.7 (0.1) | -0.1 (-0.4, 0.2) | 0.555 | 5 (0.2) | 0.2 (-0.6, 1.1) | 0.614 | 9.7 (0.1) | -0.2 (-0.4, 0) | 0.109 |
| University | 16 | 7% | 8.4 (0.7) | -0.3 (-2, 1.4) | 0.739 | 9.5 (0.5) | 0.5 (-0.7, 1.7) | 0.446 | 9.8 (0.2) | 0 (-0.5, 0.5) | 0.921 | 5.7 (0.5) | 0.9 (-0.4, 2.2) | 0.165 | 9.9 (0.1) | 0 (-0.3, 0.4) | 0.841 |
| **Occupation** |  |  |  |  |  |  |  |  |  |  |  |  |  |  |  |  |  |
| Housewife | 196 | 89% | 8.6 (0.2) | ref |  | 9.2 (0.1) | ref |  | 9.8 (0.1) | ref |  | 4.7 (0.1) | ref |  | 9.8 (0) | ref |  |
| Other | 24 | 11% | 8.6 (0.5) | 0 (-1.2, 1.1) | 0.938 | 9.4 (0.4) | 0.3 (-0.6, 1.1) | 0.525 | 9.7 (0.2) | 0 (-0.3, 0.3) | 0.906 | 4.8 (0.4) | 0.1 (-0.8, 1) | 0.790 | 9.9 (0.1) | 0.1 (-0.1, 0.3) | 0.360 |
| **Wealth index** |  |  |  |  |  |  |  |  |  |  |  |  |  |  |  |  |  |
| Lowest | 44 | 20% | 8.9 (0.4) | ref |  | 9.1 (0.3) | ref |  | 9.8 (0.1) | ref |  | 4.3 (0.3) | ref |  | 9.7 (0.1) | ref |  |
| Second | 46 | 21% | 8.6 (0.4) | -0.3 (-1.4, 0.7) | 0.533 | 9.2 (0.3) | 0.1 (-0.7, 0.8) | 0.851 | 9.8 (0.1) | 0 (-0.3, 0.3) | 0.884 | 4.5 (0.3) | 0.2 (-0.7, 1) | 0.720 | 9.8 (0.1) | 0.1 (-0.1, 0.3) | 0.446 |
| Middle | 45 | 20% | 8.4 (0.4) | -0.6 (-1.6, 0.5) | 0.303 | 9.3 (0.3) | 0.2 (-0.6, 1) | 0.589 | 9.8 (0.1) | 0 (-0.3, 0.3) | 0.871 | 4.8 (0.3) | 0.5 (-0.4, 1.3) | 0.280 | 9.8 (0.1) | 0.1 (-0.1, 0.3) | 0.217 |
| Fourth | 44 | 20% | 9 (0.4) | 0.1 (-1.1, 1.2) | 0.880 | 9.2 (0.3) | 0.2 (-0.7, 1) | 0.688 | 9.7 (0.1) | -0.1 (-0.4, 0.2) | 0.508 | 4.8 (0.3) | 0.5 (-0.4, 1.4) | 0.254 | 9.9 (0.1) | 0.2 (0, 0.4) | 0.056 |
| Highest | 41 | 19% | 8 (0.4) | -0.9 (-2.1, 0.3) | 0.130 | 9.1 (0.3) | 0 (-0.8, 0.9) | 0.912 | 9.8 (0.1) | 0 (-0.4, 0.3) | 0.927 | 5.2 (0.3) | 0.9 (-0.1, 1.8) | 0.072 | 9.8 (0.1) | 0.1 (-0.1, 0.4) | 0.326 |
| **Breastfeeding practice** | |  |  |  |  |  |  |  |  |  |  |  |  |  |  |  |  |
| Partial breastfeeding | 38 | 17% | 9.1 (0.4) | ref |  | 9.3 (0.3) | ref |  | 9.6 (0.1) | ref |  | 4.9 (0.3) | ref |  | 9.8 (0.1) | ref |  |
| Exclusive breastfeeding | 182 | 83% | 8.5 (0.2) | -0.5 (-1.5, 0.4) | 0.246 | 9.1 (0.1) | -0.2 (-0.9, 0.5) | 0.564 | 9.8 (0.1) | 0.2 (-0.1, 0.5) | 0.131 | 4.7 (0.1) | -0.2 (-1, 0.5) | 0.525 | 9.8 (0) | 0 (-0.2, 0.2) | 0.715 |
| **Parity** |  |  |  |  |  |  |  |  |  |  |  |  |  |  |  |  |  |
| Primiparous | 72 | 33% | 8.6 (0.4) | ref |  | 9.1 (0.3) | ref |  | 9.8 (0.1) | ref |  | 4.9 (0.3) | ref |  | 9.9 (0.1) | ref |  |
| Multiparous | 148 | 67% | 8.6 (0.2) | 0 (-0.9, 0.9) | 0.953 | 9.2 (0.2) | 0.1 (-0.5, 0.8) | 0.713 | 9.7 (0.1) | -0.1 (-0.4, 0.1) | 0.370 | 4.6 (0.2) | -0.2 (-0.9, 0.5) | 0.550 | **9.7 (0)** | **-0.2 (-0.4, -0.1)** | **0.010** |
| **Family size** |  |  |  |  |  |  |  |  |  |  |  |  |  |  |  |  |  |
| 3-4 | 86 | 39% | 8.6 (0.3) | ref |  | 9.5 (0.2) | ref |  | 9.8 (0.1) | ref |  | 5.1 (0.2) | ref |  | 9.8 (0.1) | ref |  |
| 5-6 | 80 | 36% | 8.7 (0.3) | 0.2 (-0.7, 1) | 0.682 | 8.9 (0.2) | -0.6 (-1.1, 0) | 0.071 | 9.8 (0.1) | 0 (-0.3, 0.2) | 0.813 | 4.6 (0.2) | -0.5 (-1.1, 0.2) | 0.171 | 9.7 (0.1) | -0.2 (-0.3, 0) | 0.067 |
| >7 | 54 | 25% | 8.5 (0.3) | -0.1 (-1, 0.8) | 0.902 | 9.1 (0.3) | -0.4 (-1.1, 0.3) | 0.228 | 9.7 (0.1) | -0.1 (-0.4, 0.2) | 0.476 | **4.3 (0.3)** | **-0.7 (-1.5, 0)** | **0.042** | 9.8 (0.1) | 0 (-0.2, 0.1) | 0.637 |

Bold values indicate statistical significance at p<0.05. Analysis was conducted using multiple linear regression (full model), with adherence scores as quantitative outcomes and sociodemographic characteristics as categorical variables.

Supplementary Table 2. Summary of Nutrient Intake and Probability of Adequacy (PA) Based on Estimated Average Requirements (EAR)

| **Nutrient** | **Median (IQR)** | **EAR** | **Mean PA** | **SD** |
| --- | --- | --- | --- | --- |
| Vitamin A (RAE) | 397 (275 - 560) | 450 | 0.40 | 0.49 |
| Thiamin (mg) | 1.0 (0.8 - 1.2) | 1.2 | 0.26 | 0.44 |
| Riboflavin (mg) | 1.2 (1 - 1.4) | 1.3 | 0.30 | 0.46 |
| Niacin (mg) | 10.4 (8.4 - 13.1) | 13.0 | 0.26 | 0.44 |
| Vitamin B6 (mg) | 1.1 (0.9 - 1.3) | 1.7 | 0.07 | 0.25 |
| Folate (µg) | 298 (246 - 383) | 450 | 0.12 | 0.32 |
| Vitamin B12 (µg) | 2.3 (1.7 - 3) | 2.4 | 0.45 | 0.50 |
| Vitamin C (mg) | 31.1 (21.3 - 43) | 58 | 0.07 | 0.25 |
| Iron (mg) | 11.4 (9.9 - 14.5) | 11.7 | 0.49 | 0.50 |
| Zinc (mg) | 9.3 (7.8 - 11.2) | 7 | 0.86 | 0.35 |
| Calcium (mg) | 496 (402 - 598) | 800 | 0.04 | 0.19 |
| Mean Total PA score (max. 11) |  |  | 3.35 | 2.43 |

The Probability of Adequacy (PA) was calculated for each individual and nutrient using the Estimated Average Requirement (EAR). A PA score of 1 was assigned if the individual's intake met or exceeded the EAR, and 0 if the intake was below the EAR. The Total PA Score represents the sum of PA values across all assessed nutrients for each individual, providing an overall measure of nutrient adequacy.

Supplementary Table 3a. Beta Coefficients (95% CI) Between Adherence Scores and Nutrition Biomarker: Mother Nutrition Biomarker

| **Component** | **Mother** | | | | |
| --- | --- | --- | --- | --- | --- |
|  | **Obesity** | **Blood marker** | | | |
|  | BMI | Iron | Zinc | Vit A | Vit B12 |
|  | kg/m^2^ | μg/L | µmol/L | μg/L | pmol/L |
| Mean | 24.9 | 35.9 | 10.4 | 1.4 | 298.4 |
| SD | 3.7 | 29.7 | 1.3 | 0.4 | 115.2 |
| Total score | 0 (-0.08 - 0.07) | 0.05 (-0.58 - 0.69) | 0 (-0.03 - 0.02) | 0 (-0.01 - 0.01) | 1.98 (-0.15 - 4.11) |
| Vegetables^a^ | 0.25 (-0.12, 0.62) | 0.84 (-2.33, 4.01) | -0.01 (-0.15, 0.13) | 0.04 (-0.01, 0.08) | -4.98 (-15.86, 5.90) |
| Fruits^a^ | -0.00 (-0.15, 0.14) | 0.56 (-0.68, 1.80) | -0.03 (-0.08, 0.02) | 0.00 (-0.01, 0.02) | **6.07 (1.76, 10.38)** |
| Protein foods^a^ | -0.06 (-0.39, 0.26) | 1.20 (-1.80, 4.20) | -0.01 (-0.14, 0.13) | 0.01 (-0.03, 0.05) | 2.09 (-7.89, 12.07) |
| Starchy staples^b^ | -0.15 (-0.42, 0.12) | -1.45 (-3.92, 1.02) | **0.12 (0.01, 0.22)** | -0.01 (-0.04, 0.02) | -2.51 (-10.44, 5.43) |
| Sugar^b^ | -0.04 (-0.33, 0.24) | **-2.90 (-5.55, -0.25)** | 0.10 (-0.01, 0.22) | -0.00 (-0.03, 0.03) | -3.39 (-11.74, 4.95) |
| Salt^b^ | 0.15 (-0.15, 0.44) | 1.15 (-1.32, 3.63) | **-0.11 (-0.22, -0.00)** | -0.03 (-0.06, 0.00) | -0.58 (-9.27, 8.10) |
| Fats and oils^b^ | -0.50 (-1.28, 0.28) | 1.35 (-6.23, 8.94) | 0.03 (-0.30, 0.37) | -0.04 (-0.13, 0.06) | **-23.08 (-45.70, -0.45)** |
| Water^a^ | 0.08 (-0.20, 0.35) | -1.02 (-3.35, 1.32) | 0.05 (-0.05, 0.15) | -0.02 (-0.05, 0.01) | -0.54 (-8.50, 7.42) |
| Limit coffee^b^ | 0.29 (-0.77, 1.35) | 1.18 (-7.70, 10.06) | -0.11 (-0.50, 0.28) | 0.10 (-0.02, 0.21) | 1.36 (-30.07, 32.78) |
| Have breakfast^a^ | -0.15 (-0.35, 0.05) | 0.19 (-1.58, 1.97) | -0.01 (-0.09, 0.07) | -0.00 (-0.03, 0.02) | 5.55 (-0.52, 11.63) |

a) Adequacy component, b) Moderation component. Bold values indicate statistical significance at p<0.05. Adherence scores (1–10) were analysed using multivariate regression without standardising effect sizes. Covariates for the analysis included maternal age, total energy intake, and nutrient-specific intake (e.g., dietary iron for serum ferritin) for maternal blood biomarkers. Nutritional status biomarker values were adjusted for inflammation markers.

Supplementary Table 3b. Beta Coefficients (95% CI) Between Adherence Scores and Nutrition Biomarker: Breastmilk Composition

| **Component** | **Mother** | | | | |
| --- | --- | --- | --- | --- | --- |
|  | **Breastmilk** | | | | |
|  | Volume | Iron | Zinc | Vit A | Vit B12 |
|  | ml/d | μg/L | μg/L | μg/L | pmol/L |
| Mean | 680 | 0.2 | 0.9 | 0.4 | 242.1 |
| SD | 219 | 0.2 | 0.5 | 0.2 | 154.6 |
| Total score | 2.28 (-1.2 - 5.76) | 0 (0 - 0.01) | 0 (-0.01 - 0.01) | 0.01 (-0.00 - 0.01) | 1.79 (-1.21 - 4.78) |
| Vegetables^a^ | 0.04 (-17.64, 17.73) | 0.00 (-0.02, 0.02) | -0.04 (-0.09, 0.01) | 0.02 (-0.00, 0.05) | -2.32 (-17.52, 12.89) |
| Fruits^a^ | 1.12 (-5.99, 8.23) | 0.00 (-0.00, 0.01) | 0.00 (-0.02, 0.02) | 0.01 (-0.00, 0.02) | 3.69 (-2.35, 9.73) |
| Protein foods^a^ | 13.33 (-2.25, 28.90) | 0.01 (-0.01, 0.03) | -0.00 (-0.05, 0.04) | -0.00 (-0.02, 0.02) | 1.08 (-13.13, 15.30) |
| Starchy staples^b^ | -3.83 (-16.91, 9.24) | -0.01 (-0.02, 0.00) | 0.00 (-0.03, 0.04) | 0.01 (-0.01, 0.02) | -7.60 (-18.71, 3.52) |
| Sugar^b^ | -1.88 (-15.55, 11.79) | -0.01 (-0.03, 0.01) | -0.02 (-0.06, 0.02) | 0.01 (-0.01, 0.03) | -0.45 (-12.04, 11.14) |
| Salt^b^ | 10.70 (-3.60, 25.01) | 0.00 (-0.02, 0.02) | 0.00 (-0.04, 0.04) | -0.01 (-0.03, 0.01) | 2.73 (-9.40, 14.85) |
| Fats and oils^b^ | 22.04 (-15.28, 59.36) | -0.02 (-0.06, 0.02) | -0.02 (-0.12, 0.09) | -0.04 (-0.09, 0.01) | **-36.71 (-69.13, -4.29)** |
| Water^a^ | -5.29 (-18.43, 7.84) | -0.00 (-0.02, 0.01) | 0.01 (-0.03, 0.05) | 0.00 (-0.01, 0.02) | -4.65 (-16.08, 6.78) |
| Limit coffee^b^ | **-55.73 (-106.76, -4.70)** | 0.02 (-0.03, 0.08) | 0.07 (-0.06, 0.21) | 0.01 (-0.06, 0.08) | 2.63 (-40.35, 45.61) |
| Have breakfast^a^ | 7.07 (-2.67, 16.80) | 0.00 (-0.01, 0.01) | -0.00 (-0.03, 0.03) | 0.00 (-0.01, 0.02) | 7.08 (-1.40, 15.56) |

a) Adequacy component, b) Moderation component. Bold values indicate statistical significance at p<0.05. Adherence scores (1–10) were analysed using multivariate regression without standardising effect sizes. Covariates for the analysis included milk volume, exclusive breastfeeding status, and nutrient-specific intake for breastmilk composition. Nutritional status biomarker values were adjusted for inflammation markers.

Supplementary Table 3c. Beta Coefficients (95% CI) Between Adherence Scores and Nutrition Biomarker: Infant Nutrition Biomarker

| **Component** | **Infant** | | | | | | |
| --- | --- | --- | --- | --- | --- | --- | --- |
|  | **Growth** | | | **Blood marker** | | | |
|  | WAZ | LAZ | WLZ | Iron | Zinc | Vit A | Vit B12 |
|  | z-score | z-score | z-score | μg/L | µmol/L | μg/L | pmol/L |
| Mean | -0.60 | -0.69 | 0.08 | 46.5 | 11.3 | 1.0 | 258.5 |
| SD | 0.86 | 0.96 | 1.01 | 35.6 | 2.0 | 0.2 | 138.4 |
| Total score | -0.01 (-0.03 - 0.01) | -0.01 (-0.04 - 0.02) | -0.01 (-0.04 - 0.02) | -0.37 (-1.23 - 0.49) | -0.02 (-0.07 - 0.03) | 0 (-0.01 - 0) | 1.27 (-1.41 - 3.96) |
| Vegetables^a^ | 0.03 (-0.06, 0.12) | 0.03 (-0.12, 0.19) | 0.06 (-0.09, 0.22) | 2.82 (-1.06, 6.70) | 0.01 (-0.21, 0.24) | 0.00 (-0.02, 0.03) | -2.41 (-15.75, 10.94) |
| Fruits^a^ | 0.00 (-0.04, 0.04) | 0.00 (-0.06, 0.07) | -0.04 (-0.11, 0.03) | **-1.85 (-3.45, -0.24)** | -0.07 (-0.16, 0.02) | -0.00 (-0.01, 0.01) | 2.38 (-3.06, 7.81) |
| Protein foods^a^ | 0.01 (-0.08, 0.10) | 0.08 (-0.10, 0.25) | 0.08 (-0.10, 0.26) | -3.92 (-7.71, -0.12) | -0.06 (-0.29, 0.17) | 0.01 (-0.02, 0.03) | -0.64 (-13.08, 11.79) |
| Starchy staples^b^ | -0.03 (-0.10, 0.05) | -0.02 (-0.15, 0.11) | -0.09 (-0.23, 0.04) | 1.79 (-1.27, 4.85) | 0.09 (-0.08, 0.26) | -0.01 (-0.03, 0.01) | -1.65 (-11.43, 8.12) |
| Sugar^b^ | 0.02 (-0.06, 0.10) | -0.04 (-0.18, 0.10) | -0.02 (-0.16, 0.12) | 1.95 (-1.49, 5.39) | 0.13 (-0.07, 0.33) | -0.01 (-0.03, 0.01) | 0.55 (-9.72, 10.82) |
| Salt^b^ | -0.01 (-0.09, 0.06) | -0.10 (-0.23, 0.02) | 0.01 (-0.12, 0.14) | -3.00 (-6.08, 0.09) | 0.02 (-0.16, 0.19) | -0.00 (-0.02, 0.02) | -3.65 (-14.16, 6.86) |
| Fats and oils^b^ | 0.07 (-0.18, 0.32) | -0.04 (-0.55, 0.46) | 0.06 (-0.45, 0.58) | -8.02 (-18.25, 2.22) | 0.08 (-0.50, 0.66) | -0.01 (-0.08, 0.05) | -8.94 (-37.93, 20.04) |
| Water^a^ | -0.01 (-0.08, 0.05) | -0.03 (-0.13, 0.08) | 0.02 (-0.09, 0.13) | 1.06 (-1.88, 3.99) | -0.09 (-0.27, 0.08) | 0.01 (-0.01, 0.03) | 4.12 (-6.47, 14.71) |
| Limit coffee^b^ | **-0.37 (-0.65, -0.10)** | -0.05 (-0.45, 0.34) | -0.38 (-0.79, 0.02) | 4.41 (-7.13, 15.95) | -0.04 (-0.67, 0.58) | **-0.10 (-0.17, -0.03)** | 34.42 (-1.78, 70.63) |
| Have breakfast^a^ | -0.03 (-0.09, 0.02) | -0.04 (-0.14, 0.06) | -0.10 (-0.20, 0.00) | 0.67 (-1.59, 2.93) | -0.03 (-0.17, 0.11) | 0.00 (-0.01, 0.02) | 0.54 (-7.56, 8.65) |

a) Adequacy component, b) Moderation component. Bold values indicate statistical significance at p<0.05. Adherence scores (1–10) were analysed using multivariate regression without standardising effect sizes. Covariates for the analysis included exclusive breastfeeding, birth size (length and weight), infant inflammation markers, infant energy intake for infant growth indicators; and maternal/infant nutrient-specific intake for infant blood biomarkers. Nutritional status biomarker values were adjusted for inflammation markers.
